# FDA approval of crovalimab: a milestone in paroxysmal nocturnal hemoglobinuria treatment

**DOI:** 10.1097/MS9.0000000000002848

**Published:** 2025-01-09

**Authors:** Diya Anwar, Asim Ali Khan, Hooriya Ahmed, Syeda Rashmeen, Solay Farhat, Syeda Shahnoor, Abdul Moiz Khan

**Affiliations:** a Department of Internal Medicine, Ayub Medical College, Abbottabad, Pakistan; b Department of Internal Medicine, Rawalpindi Medical University, Rawalpindi, Pakistan; c Department of Internal Medicine, Jinnah Sindh Medical University, Karachi, Pakistan; d Faculty of Medical Sciences, Lebanese University, Beirut, Lebanon; e Department of Internal Medicine, Dow University of Health Sciences, Karachi, Pakistan

*Dear Editor*,

Paroxysmal nocturnal hemoglobinuria (PNH) is a rare hematopoietic stem cell disorder characterized by hemolytic anemia, thrombosis, smooth muscle dystonia, and bone marrow failure. The global prevalence of PNH is as high as 38 individuals per million, with higher prevalence in certain Asian countries like Japan, Korea, and China compared to the West^[1]^. PNH arises from a deficiency in complement inhibitors, specifically due to mutations in the phosphatidylinositol N-acetylglucosaminyltransferase subunit A (PIGA) gene, leading to the absence of glycosylphosphatidylinositol (GPI)-anchored proteins, such as CD55 and CD59, which protect red blood cells from complement-mediated destruction^[2]^. The resulting uncontrolled complement activation causes intravascular hemolysis, releasing free hemoglobin that depletes nitric oxide, contributing to smooth muscle dystonia. Additionally, chronic hemolysis and complement overactivation trigger platelet activation and increase the risk of thrombotic events, while immune-mediated damage to hematopoietic stem cells can result in bone marrow failure^[1]^.

PNH symptoms vary widely and severely impact patients’ quality of life. Chronic destruction of red blood cells results in severe anemia, manifesting as fatigue, shortness of breath, and paleness^[3]^. Patients frequently suffer from hemoglobinuria, which causes dark or hematic urine. Additionally, hemolytic anemia often necessitates multiple blood transfusions, which can elevate the risk of iron overload and associated complications^[4]^. Bone marrow failure in PNH leads to pancytopenia, making patients more susceptible to infections, bruising, and bleeding^[5]^. The combination of chronic hemolysis, frequent transfusions, and risks of thrombosis and bone marrow failure creates a significant physical and emotional burden, affecting overall quality of life^[1]^.

## Current treatment options

Up until now, five drugs had received FDA approvals for treating PNH, either as add-on therapies or as various complement inhibitors: Eculizumab, **Danicopan**, Ravulizumab, Pegcetacoplan, and Iptacopan. Table 1 summarizes the details of these FDA-approved drugs for PNH.

**Table 1:** Summary of FDA approved drugs for Paroxysmal nocturnal hemoglobinuria.

Drug	Date of approval	Class	Mode of administration	Clinical Trails
Eculizumab	March, 2007	C5 complement inhibitor	Intravenously every 2 weeks	Phase 3 TRIUMPH Trial (NCT00122330) & SHEPHERD Trial (NCT00130000)
Ravulizumab	December, 2018	C5 complement inhibitor	Intravenously every 8 weeks	Phase 3 Trials (NCT02946463 & NCT03056040)
Pegcetacoplan	May, 2021	C3 complement inhibitor	Subcutaneously twice weekly	Phase 3 PEGASUS trial (NCT03500549)
Iptacopan	December, 2023	Factor B inhibitor	Orally twice daily	Phase 3 APPLY-PNH trial (NCT04558918) & APPOINT-PNH (NCT04820530)
Danicopan	April, 2024	Factor D inhibitor	Orally thrice daily	Phase 3 ALPHA trial (NCT04469465)

Eculizumab and Ravulizumab, both C5 inhibitors, block the terminal complement cascade by preventing C5 cleavage, inhibiting the formation of the membrane attack complex (MAC). While effective in reducing hemolysis and thromboembolic events, both require intravenous administration, with Ravulizumab offering less frequent dosing. Pegcetacoplan, a C3 inhibitor, prevents both intravascular and extravascular hemolysis but requires infection monitoring due to broader complement suppression. Danicopan, an oral factor D inhibitor, complements C5 inhibitors to prevent breakthrough hemolysis. Iptacopan, a factor B inhibitor, targets the alternative pathway, offering a more convenient oral option.

While effective in disease management, these treatments have notable shortcomings, such as incomplete complement inhibition, breakthrough hemolysis, inadequate control of bone marrow failure, and the risk of clonal evolution^[6]^. Addressing these limitations requires the development of advanced complement inhibitors and improved drugs that offer comprehensive management of PNH, while also being easy to administer and practical.

## Crovalimab: A promising new treatment option for PNH

Crovalimab is a new potential drug being explored to address the limitations of previously available treatments. In February 2024, it received initial approval in China for treating both adolescents and adults with PNH, followed by approval in Japan in March 2024^[7]^. The latest development is the approval of Crovalimab-akkz (Piasky) by the FDA on 24 June 2024^[8]^. Crovalimab, developed by Chugai Pharmaceutical in collaboration with Roche, is a humanized monoclonal antibody targeting complement component C5. It works by inhibiting complement C5, thereby hindering its transformation into C5a and C5b. This inhibition prevents the formation of the membrane attack complex (MAC) and reduces intravascular hemolysis^[9]^.

## Clinical trials assessing crovalimab efficacy

The drug Crovalimab was approved after the Phase 3 COMMODORE 2 trial, which enrolled 204 PNH patients without prior disease-specific treatment^[10]^. Of these, 135 patients received Crovalimab and 69 patients received Eculizumab, with 38.7% having a history of aplastic anemia and 5.9% myelodysplastic syndrome. Results showed that about two-thirds of the patients on Crovalimab therapy did not require any sort of further blood transfusions, and nearly 80% experienced no hemolysis. Most completed the 24-week treatment and entered the extension phase. This trial effectively demonstrates the non-inferiority of Crovalimab to the already established treatment, Eculizumab, in managing PNH. After the completion of trial, 84% of patients in the COMMODORE 2 trial expressed a preference for Crovalimab over Eculizumab. The primary reasons for this preference included the easier method of administration, fewer hospital visits required, and a shorter time needed to administer the treatment^[10]^.

Likewise, the Phase 3 COMMODORE 3 trial from China showed that 78.7% of patients achieved hemolysis control, and 51% avoided transfusions^[11]^. While common side effects included respiratory tract infections, infusion-related reactions, and type 3 hypersensitivity reactions, no treatment discontinuations occurred due to side effects in either trial, demonstrating Crovalimab’s efficacy and tolerability. The COMMODORE 1 trial results also demonstrated a better safety profile for Crovalimab, providing sustained complement inhibition, disease control, and showing that 85% of patients preferred it over Eculizumab^[12]^. The findings from clinical trials evaluating Crovalimab efficacy are outlined in Table 2.

**Table 2:** Summary of clinical trials evaluating Crovalimab efficacy.

Trial	Design	Number of participants	Intervention	Control	Duration	Results
COMMODORE 1^[11]^	Phase 3, randomized, multicentral trial	89	Crovalimab	Eculizumab	24 weeks	Crovalimab maintained robust complement activity inhibition, achieved disease control, with 85% patient preference over eculizumab.
COMMODORE 2^[9]^	Phase 3, randomized, multicentral trial	204	Crovalimab	Eculizumab	24 weeks	Crovalimab matched eculizumab in controlling hemolysis and reducing transfusions, with fewer breakthrough events, and stabilized hemoglobin levels.
COMMODORE 3^[10]^	Phase 3, single arm, multicentral trial	51	Crovalimab	none	24 weeks	Crovalimab effectively controlled hemolysis in about 78.7% of patients and markedly decreased the requirement for transfusions compared to baseline.

## Recommended dosage

Crovalimab represents the first complete treatment for PNH, administered monthly via subcutaneous injection, offering the convenience of self-administration at home. The prescribed dosing schedule starts with an initial 1000-1500 mg intravenous (IV) infusion on Day 1, followed by four weekly 340 mg subcutaneous injections on Days 2, 8, 15, and 22. The maintenance dose of 680-1020 mg, begins on Day 29 and is administered every 4 weeks via subcutaneous injection thereafter^[8]^.

### Recommendations

This approval marks a significant breakthrough in hematological sciences and has the potential to transform the lives of patients suffering from PNH. Clinicians across the healthcare spectrum should be promptly informed about Crovalimab, ensuring they recognize it as a viable treatment option. With its promising efficacy, it is crucial that this drug is made available on a large scale to benefit as many patients as possible. Global accessibility will require partnerships between Roche and local health authorities to streamline distribution and integrate Crovalimab into national healthcare plans. Pricing strategies must ensure affordability and equitable access, especially in resource-limited settings. Collaborations with patient advocacy groups will be crucial for raising awareness, promoting timely diagnosis, and facilitating treatment access.

Additionally, treatment guidelines and policies need to be updated to include Crovalimab as a standard therapy option. Furthermore, training programs and workshops should be organized for healthcare providers to familiarize them with the administration and management of Crovalimab. Patient education initiatives are also essential to ensure adherence and optimize outcomes. However, while this approval is a milestone, we must continue to invest in research and development to further enhance treatment outcomes for those suffering from paroxysmal nocturnal hemoglobinuria, always striving for better patient care. Continued surveillance and real-world studies will be necessary to monitor long-term efficacy and safety, ensuring that the benefits observed in clinical trials translate effectively into everyday clinical practice.

## Limitations

C5 inhibitors, including Crovalimab, have notable limitations, such as non-responsiveness in patients with C5 polymorphisms and breakthrough hemolysis triggered by infections or complement-amplifying conditions. Additionally, Crovalimab therapy may face challenges related to extravascular clearance due to C3b accumulation. Patients transitioning from other C5 inhibitors to Crovalimab are also at risk of developing Drug-Target-Drug Complexes (DTDCs), which could impact treatment effectiveness. Addressing these challenges will be essential to optimizing therapy and ensuring better outcomes for PNH management.

## Conclusion

PNH profoundly impacts patients with severe anemia, hemolysis, and heightened thrombotic risks, necessitating frequent transfusions and posing significant complications like iron overload and bone marrow failure. While current treatments have limitations, Crovalimab represents a promising advance with improved disease control, patient preference, and safety. Future studies should explore long-term efficacy, real-world outcomes, and comparative effectiveness. Research on personalized approaches, combination therapies, and global collaborations will be key to optimizing care and expanding access.

## Data Availability

Not Applicable.
